# Selective Pre-leaching of Tellurium From Telluride-Type Gold Concentrate

**DOI:** 10.3389/fchem.2021.593888

**Published:** 2021-03-25

**Authors:** Wei Yang, Xuechen Lan, Qian Wang, Ping Dong, Gang Wang

**Affiliations:** ^1^ School of Resource Engineering, Xi'an University of Architecture and Technology, Xi’an, China; ^2^ Key Laboratory of Gold and Resources in Shaanxi Province, Xi’an, China

**Keywords:** telluride-type gold concentrate, cooperative leaching, pre-separation, tellurium, leaching rate

## Abstract

With a telluride-type gold ore flotation concentrate as the research object, the Na_2_S + NaOH collaborative leaching process was applied to selectively separate tellurium before the cyanide leaching of gold and silver. The effects of process parameters including the type of leaching agent, the amount of leaching agent, liquid-solid ratio, leaching temperature, and leaching time on the leaching rate of tellurium were investigated. The results showed that the tellurium leaching rate could reach 78.14% under the optimum conditions of −0.038 mm (95%) grinding fineness, 80 g/L Na_2_S concentration, 30 g/L NaOH concentration, 4:1 liquid-solid ratio, 80°C leaching temperature and 3 h′s leaching time. The kinetic analysis showed that the leaching process of tellurium from telluride-type gold concentrate was a mixed type of chemical reaction control and diﬀusion control. The grain parameter in the leaching process was 0.26263 and the apparent activation energy E = 17.12 kJ/mol. Tellurium could be pre-leached from the telluride-type gold flotation concentrate through the Na_2_S + NaOH alkaline leaching process to achieve the effective separation of tellurium from noble metals, which, when eliminating the adverse effects of telluride on the leaching of gold and silver, provides new ideas for the extraction of rare element tellurium.

## Introduction

Tellurium, a rare element, is widely used in metallurgy, chemical industry, electronics, aerospace, medical and other fields (Wang, 2011). As an additive in metallurgy, tellurium can improve the cutting properties of steel and copper, and enhance the hardness and wear resistance of "baffle alloys" (Wang, 2011); CdTe thin-film solar cells are a kind of solar cells with low price and their highest photoelectric conversion efficiency could reach 21%, holding the greatest promise for the future of thin film (Lee and Ebong, 2017; Geisthardt et al., 2015); Tellurium compounds such as ammonium trichloro(dioxo ethylene-*O*, *O*′)tellurate (AS101) have attracted much attention in cancer treatment (Sredni, 2012); besides, tellurium and its compounds have gradually become research hotspots in thermoelectric conversion, biology, and electronics (Lin et al., 2016; Ba et al., 2010; Chivers and Laitinen, 2015).

Although tellurium is widely used, its abundance in the Earth's crust is only 1 ppb, which is lower than that of the so-called “rare earth” elements, and often coexists with chalcogen elements such as gold, copper, and lead (Missen et al., 2020). Currently, tellurium comes mainly from copper-refined anode sludge from smelters, which accounts for more than 90% of its global supply (Wang, 2011; Fan et al., 2013). With the increasing demand and price of tellurium, the recovery of tellurium from tellurium-containing ores or electronic wastes has attracted researchers’ attention (Rocchetti and Beolchini, 2015; Candelise et al., 2012). The Dashuigou Bismuth Tellurium Deposit in Shimian County, Sichuan Province, China is the only independent primary tellurium deposit discovered in the world so far, so there are many studies on the recovery of tellurium from bismuth tellurium deposits (Zhang et al., 2019). The H_2_SO_4_ + FeCl_3_ oxidative leaching process was applied to leach bismuth telluride ore and the leaching rate of tellurium and bismuth could reach 95.61 and 95.77% (Shao et al., 2020). A large number of flotation experiments were carried out on bismuth tellurium deposits and the tellurium grade and recovery rate of obtained concentrate products are 9.94 and 94.81%, respectively (Yu et al., 2019). This process effectively recovered bismuth, gold, and silver, which provided a reference for processing this type of minerals. Although there are some studies on the recovery of tellurium from tellurium-bearing ores, there are few studies on the recovery of tellurium from telluride-type gold ore.

The relationship between tellurium and gold is very close. Telluride-type gold ores are the most common gold-bearing minerals and also one of the refractory gold ores (Ibers, 2009). Many studies have shown that telluride-type gold ore is difficult to dissolve in the cyanide solution, resulting in a decrease in the leaching rates of gold and silver. The currently accepted explanation is that the insoluble compound—TeO_2_ (or hydrated H_2_TeO_3_ phase) was produced during the cyanidation process, and the insoluble compound could lead to a passivation layer formed on the mineral surface, as shown in Eqs 1,2 (Jayasekera et al., 1996; Henley et al., 2001; Dyer et al., 2017).

$$\;\mathrm{AuT}e_{2}\left(s\right)+CN^{-}+8OH^{-}\to \mathrm{Au}{\left(\mathrm{CN}\right)}^{2-}+2H_{2}\mathrm{Te}O_{3}\left(s\right)+2H_{2}O+7e^{-}$$ (1)

$$H_{2}\mathrm{Te}O_{3}\left(s\right)+2OH^{-}\to \mathrm{Te}O_{3}^{2-}+2H_{2}$$ (2)

Therefore, such pretreatment methods as ultra-fine grinding (UFG), roasting, and biological leaching are often applied in dealing with telluride-type gold ores to increase gold recovery rate (Dyer, et al., 2017; Richmond, 2010). However, UFG increases the consumption of chemicals in subsequent processes; roasting produces SO_2_ which is not friendly to the environment and the composition of the minerals after roasting is complex; the biological leaching process is slow and time-consuming, and the degree of tellurium oxidation is difficult to control (Dyer, et al., 2017; Richmond, 2010). In addition to the above methods, telluride-type gold ore can also be treated by flotation process. The telluride leaching method of The Republic of Fiji is a classic combined processing and metallurgical treatment of telluride-type gold ore. This process first concentrates telluride-type gold ore, then follows by oxidative roasting, cyanide leaching, polysulfide leaching, sodium sulfite reduction, and other processes to recover tellurium and gold (Zhou and Chen, 2008). This method has a complicated process and high investment cost, and is difficult to apply to other processing plants. Previously, the author’s research team obtained tellurium-gold-silver mixed concentrates from tellurium-type gold ores typical of the Xiaoqinling area by flotation, and obtained tellurium, gold, and silver with average grades of 241.61, 90.30, and 92.74 g/t with superior recoveries of 95.42, 97.28, and 94.65%, respectively, but did not achieve the separation of tellurium from the precious metals (Yang et al., 2019). The purpose of the above methods is to remove tellurium or to recover tellurium, gold and silver at the same time. At present, there are few types of research to improve the leaching rates of gold and silver by pre-leaching tellurium.

A simple process was proposed to separate tellurium from telluride-type gold concentrate and the optimal conditions were explored for selective pre-leaching of tellurium from telluride-type gold concentrate. The effects of leaching agent type, leaching agent dosage, liquid-solid ratio, leaching temperature and leaching time on the tellurium leaching rate are investigated, so as to provide the theoretical and technological basis for the separation and recovery of valuable metals in telluride-type gold concentrate.

## Experimental

### Materials

**FIGURE 1:**
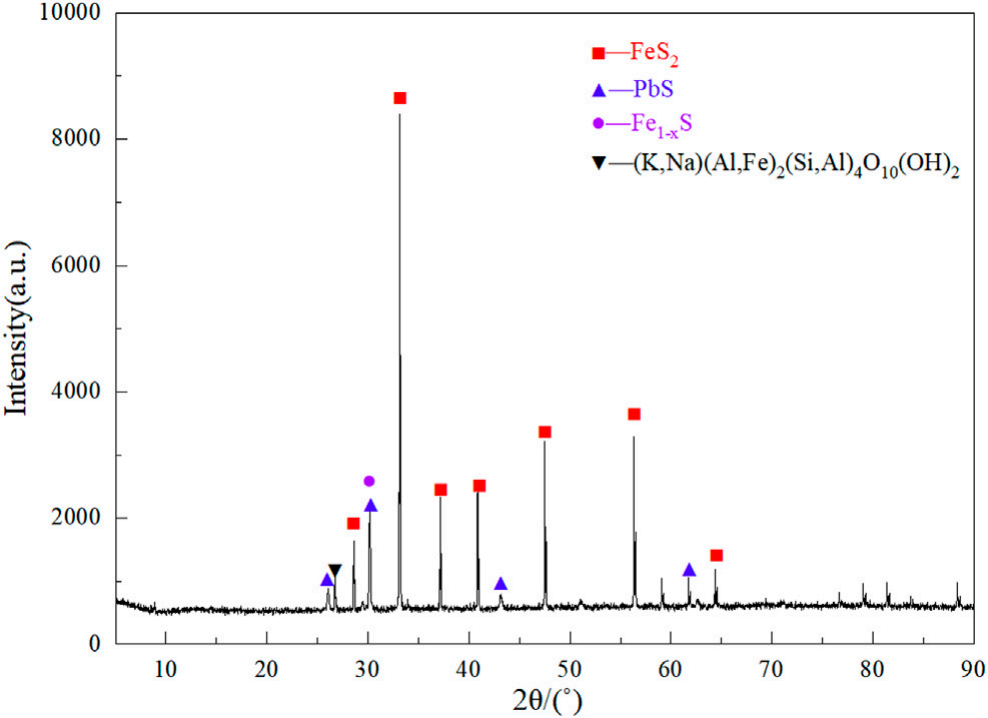
XRD diffractograms of telluride-type gold concentrate.

The sample for this experiment was taken from the telluride-type gold concentrate from the Yangping mining area in Xiaoqinling, Henan Province, China. The sample was dried, ground, and thoroughly mixed by the quarter method after it was retrieved. A small amount of the mineral sample was used for XRD analysis, and the rest was stored in a dry vessel for subsequent experiments. The chemical analysis results of the telluride-type gold concentrate have been shown in Table 1. As shown in Table 1, tellurium, gold and silver grades of 243.72, 89.30, and 93.16 g/t, respectively, and lead content 6.80%. The XRD analysis results are shown in Figure 1. The main components of the ore sample are pyrite, galena, pyrrhotite, and muscovite.

**TABLE 1:** Chemical analysis results of major elements in telluride-type gold concentrate.

Elemental	Te	Au	Ag	Pb^a^
Grade (g/t)	243.72	89.30	93.16	6.80

^a^ Means that the unit is %.

### Experimental Methods

Fifty grams telluride-type gold concentrate was weighed at a time and ground to the specified fineness in a ball mill (XMQ series ball mill) with a pulp density of 60 wt%. AR grade chemicals and deionized water were used throughout the experiment. The leaching experiment was carried out in a 250 mL beaker using a numerically controlled mechanical stirrer. In batch experiments, 50 g grinding sample was mixed with leaching agent mixture and a fixed amount of water. The pulp was leached at a stirring speed of 500 r/min and at a set temperature. The time spent in the leaching process was recorded, and the amount of water was regularly added to control the liquid-solid ratio. Finally, the leaching residue was washed five times by hot water immediately, and then dried at room temperature, weighed, sampled, and tested, and the remaining samples were kept for subsequent leaching of gold and silver. The content of tellurium in the solution was measured by ICP-MS (Agilent 7800). The leaching rate is calculated according to Eq. 3 based on the grade of the leaching residue.

$$\varepsilon =\frac{R_{0}-R}{R_{0}}\times 100\%$$ (3)

Where *ε* is the metal leaching rate (%); R_0_ and R respectively represent the grades of the element in the original ore and leach residue (g/t).

### Experimental Principle

Tellurium is mainly present in minerals or anode mud in the form of tellurium dioxide, tellurite, and tellurate, and depending on the choice of leaching agent, tellurium of different valences will undergo different reactions, and the following chemical reactions may occur during the leaching process (Zhou and Chen, 2008; Liu et al., 2020; Guo et al., 2017; Sun and Zheng, 2011; Hoffmann and Zhong, 1990).

$$\mathrm{Te}O_{2}+2\mathrm{NaOH}=Na_{2}\mathrm{Te}O_{3}+H_{2}O$$ (4)

$$Na_{2}\mathrm{Te}O_{3}+3Na_{2}S+3H_{2}O=Na_{2}\mathrm{Te}S_{3}+6\mathrm{NaOH}$$ (5)

$$Na_{2}\mathrm{Te}O_{4}+4Na_{2}S+4H_{2}O=Na_{2}\mathrm{Te}S_{4}+8\mathrm{NaOH}$$ (6)

$$Na_{2}\mathrm{Te}O_{4}+H_{2}SO_{4}=H_{2}\mathrm{Te}O_{4}+Na_{2}SO_{4}$$ (7)

$$\mathrm{Te}O_{2}+4\mathrm{HCl}=\mathrm{TeC}l_{4}+2H_{2}O$$ (8)

$$\mathrm{Te}+3Na_{2}S_{2}\to Na_{2}\mathrm{Te}S_{4}+2Na_{2}S$$ (9)

Sodium sulfide can convert insoluble tellurite into soluble thio-tellurite (Eqs 5,6). HCl can dissolve tetravalent tellurium (Eq. 8), and dilute sulfuric acid converts insoluble sodium tellurate to soluble telluric acid (Eq. 7), which, through hydrochloric acid and sulfur dioxide reduction process, or sodium sulfite reduction, alkali solution, electrolysis, and other treatments to obtain metal tellurium (Hoffmann and Zhong, 1990). The polysulfide leaching tellurium has a better leaching effect on elemental tellurium (Eq. 9), while leaching tellurium, it has an inhibitory effect on lead, copper, arsenic, etc. (Yang, 1997)

## Results and Discussion

### Effect of Leaching Agent Type

**FIGURE 2:**
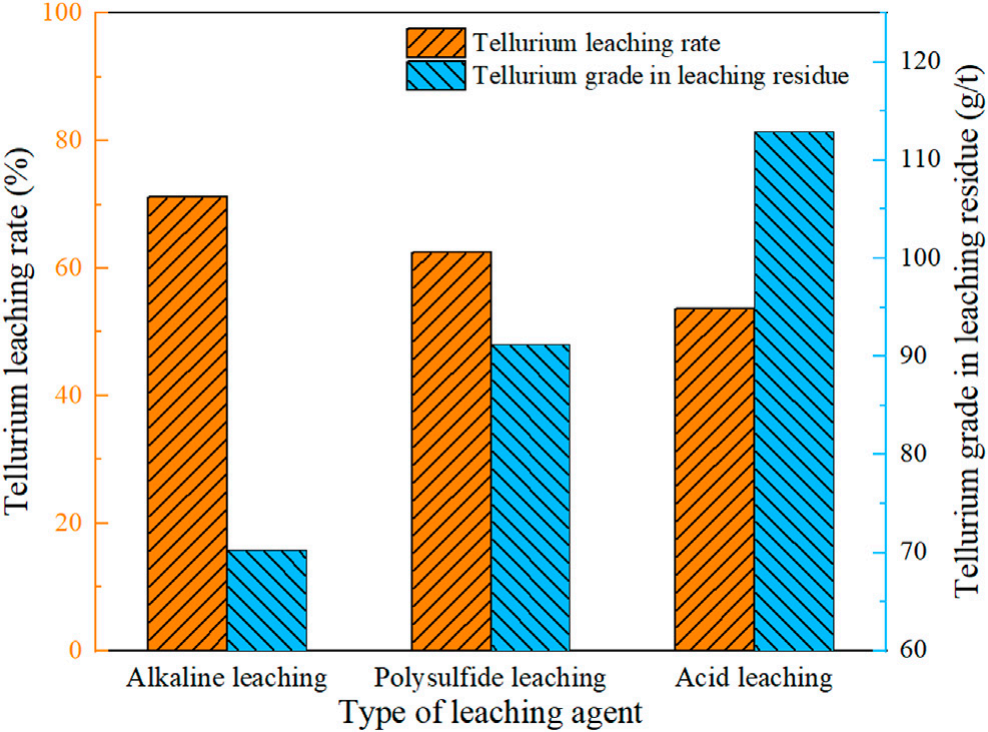
Effect of leaching agent type on tellurium leaching rate.

Figure 2 shows the effect of leaching agent type on the leaching effect of telluride-type gold concentrate. Based on experience and existing researches, the experiment was carried out as the grinding fineness was set at −0.038 mm (92%), leaching temperature at 80°C, liquid-solid ratio at 2:1, leaching time at 4 h and leaching agent dosage at 60 g/L (Na_2_S: NaOH = 2:1).

Figure 2 shows that alkaline leaching is the most effective with the tellurium leaching rate up to 71.19%. When HCl was used as the leaching agent, the leaching was the worst, with a leaching rate of 53.68%, while HCl converted the silver to insoluble silver chloride, which made it difficult for tellurium to be subsequently separated from the precious metal, and hexavalent tellurium could oxidize HCl and produce chlorine gas which can dissolve gold (Chen and Li, 2008). Compared with acid leaching and polysulfide leaching, this type of telluride-type gold concentrate has the best alkaline leaching effect, and the subsequent gold-silver leaching process is also in an alkaline environment. In summary, the Na_2_S + NaOH cooperative leaching process is adopted in the pretreatment of telluride-type gold concentrate.

### Effect of Grinding Fineness

**FIGURE 3:**
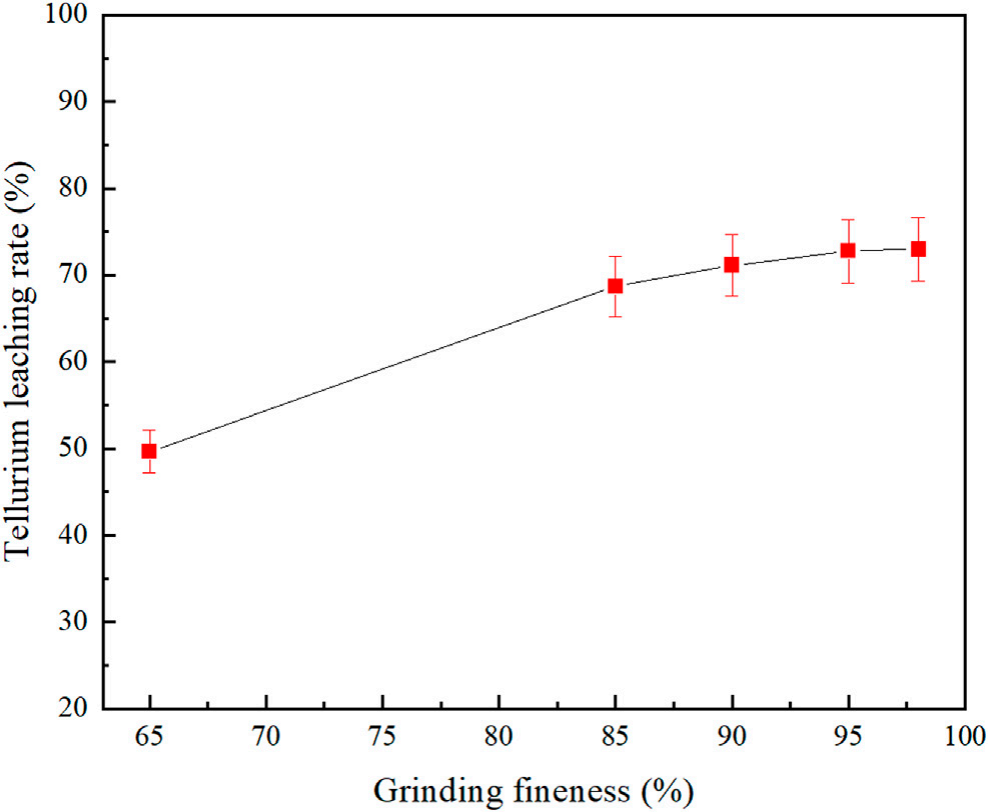
Effect of grinding fineness on tellurium leaching.

The leaching process undergoes a multiphase reaction at the solid-liquid phase boundary of the ore grains, and the phase boundary area and viscosity of the leached pulp are closely related to the grinding fineness. Therefore, in order to investigate the effect of grinding fineness on tellurium leaching rate, a grinding fineness experiment was carried out at 80°C leaching temperature, 2:1 liquid-solid ratio, 4 h’s leaching time and 60 g/L leaching agent dosage (Na_2_S: NaOH = 2:1). It can be seen from Figure 3 that the leaching rate of tellurium gradually increases with the increase in grinding fineness and then stabilizes. The experimental results are shown in Figure 3.

When grinding fineness was −0.038 mm (95%), the tellurium leaching rate reached 72.77%, while the tellurium leaching rate was almost unchanged when the grinding fineness continued to increase. This may be due to the fact that within a certain range, as the specific surface area increases with decreasing particle size (Zheng and Chen, 2014), the contact area increases between the mineral and the leaching agent, leading to the mass transfer enhanced and the leaching rate increases accordingly. However, if the grinding is too fine, it will increase the viscosity of the pulp and the diffusion resistance, and even form a mud film on the surface of the leached minerals, resulting in the slow rise or fall of the leaching rate. And the grinding time is too long, it will increase energy consumption and cost. Therefore, the grinding fineness is determined at −0.038 mm (95%).

### Effect of Leaching Agent Dosage

**FIGURE 4:**
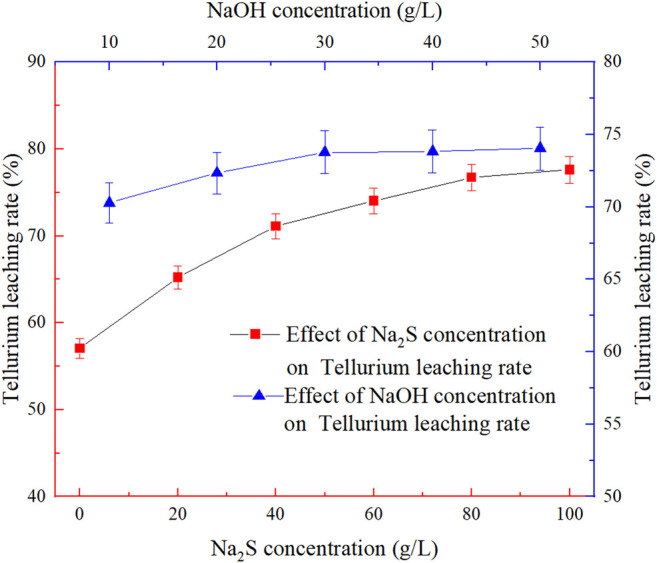
Effect of leaching agent dosage on tellurium leaching rate.

Na_2_S is the main leaching agent for tellurium leaching, and its concentration is an important factor affecting the tellurium leaching rate. When the appropriate amount of Na_2_S is used, tellurium can be selectively leached, and lead leaching can be inhibited without affecting the subsequent recovery of precious metals. The effect of Na_2_S concentration on the tellurium leaching rate was shown in Figure 4. The fixed conditions were −0.038 mm (95%) grinding fineness, 20 g/L NaOH concentration, 80°C leaching temperature, 2:1 liquid-solid ratio and 4h′s leaching time.

From Figure 4 it can be seen that the Na_2_S concentration has a greater effect on the tellurium leaching rate than the NaOH concentration, and the tellurium leaching rate increases with the increase of Na_2_S concentration and then reaches to a stable state. When the Na_2_S concentration increases from 80 to 100 g/L, the tellurium leaching rate only increases by 0.9%, so the Na_2_S concentration is finally determined to be 80 g/L considering the economic cost.

The NaOH concentration experiment was carried out as the grinding fineness was set at −0.038 mm (95%), Na_2_S concentration at 80 g/L, leaching temperature at 80°C, liquid-solid ratio at 2:1 and leaching time at 4 h, so as to investigate the effect of NaOH concentration on the tellurium leaching rate. The results are shown in Figure 4.

From Figure 4 it can also be seen that the tellurium leaching rate was 57.06% when the Na_2_S concentration was 0 g/L. It can be seen that some of the tellurium-bearing minerals were leached out by milling and NaOH addition. After the Na_2_S concentration was 80 g/L and the NaOH concentration was increased from 10 to 30 g/L, the leaching rate of tellurium hardly changes with the increase of NaOH concentration, and most of the tellurium was leached. Considering the pharmaceutical cost, a NaOH concentration of 30 g/L was determined at which the tellurium leaching rate could reach 73.78%.

### Effect of Liquid-Solid Ratio

**FIGURE 5:**
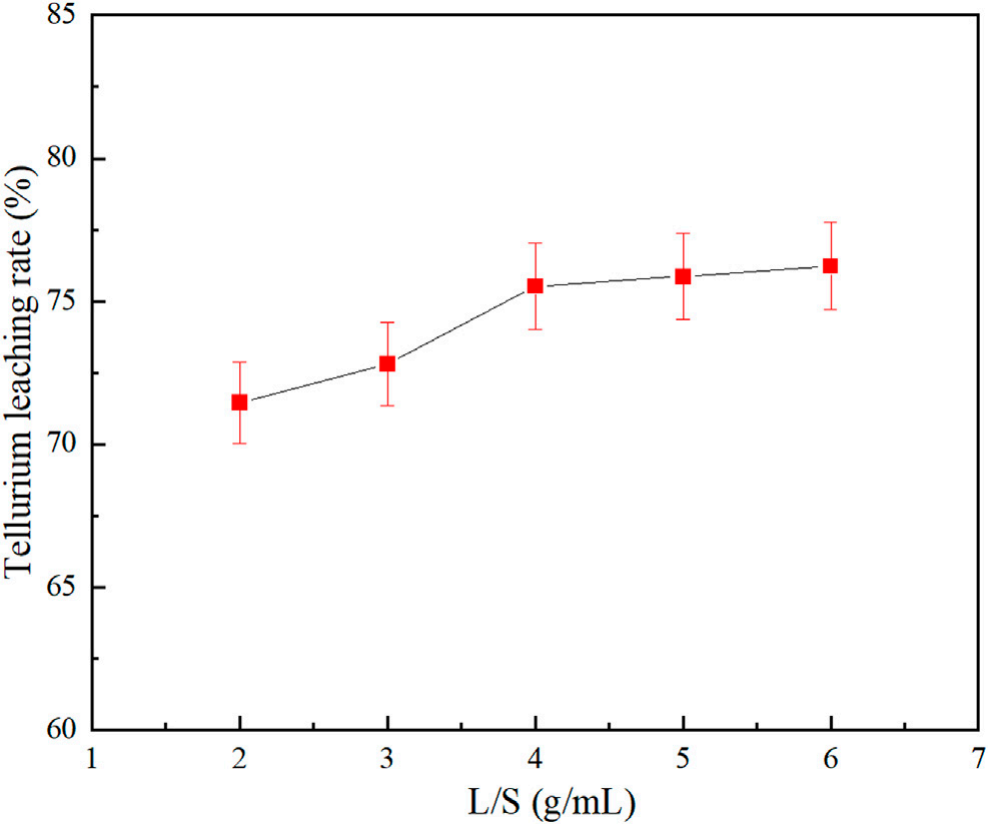
Effect of liquid-solid ratio on tellurium leaching rate.

Figure 5 shows the effect of liquid-solid ratio on the tellurium leaching rate. The experiment was carried out as the grinding fineness was set at −0.038 mm (95%), Na_2_S concentration at 80 g/L, NaOH concentration at 30 g/L, leaching temperature at 80°C and leaching time at 4 h.

As shown in Figure 5 tellurium leaching rate increased to 75.53% with increasing liquid-solid ratio and then remained almost unchanged. In the leaching process, the size of the liquid–solid ratio directly affected the liquid-solid mass transfer in the system (Aydogan et al., 2005): if the liquid–solid ratio was too small, the pulp viscosity would be high and the leaching effect would be reduced; while if the liquid–solid ratio was too large, the processing capacity of the equipment would be affected. Thus, the liquid–solid ratio was set at 4:1.

### Effect of Leaching Temperature

**FIGURE 6:**
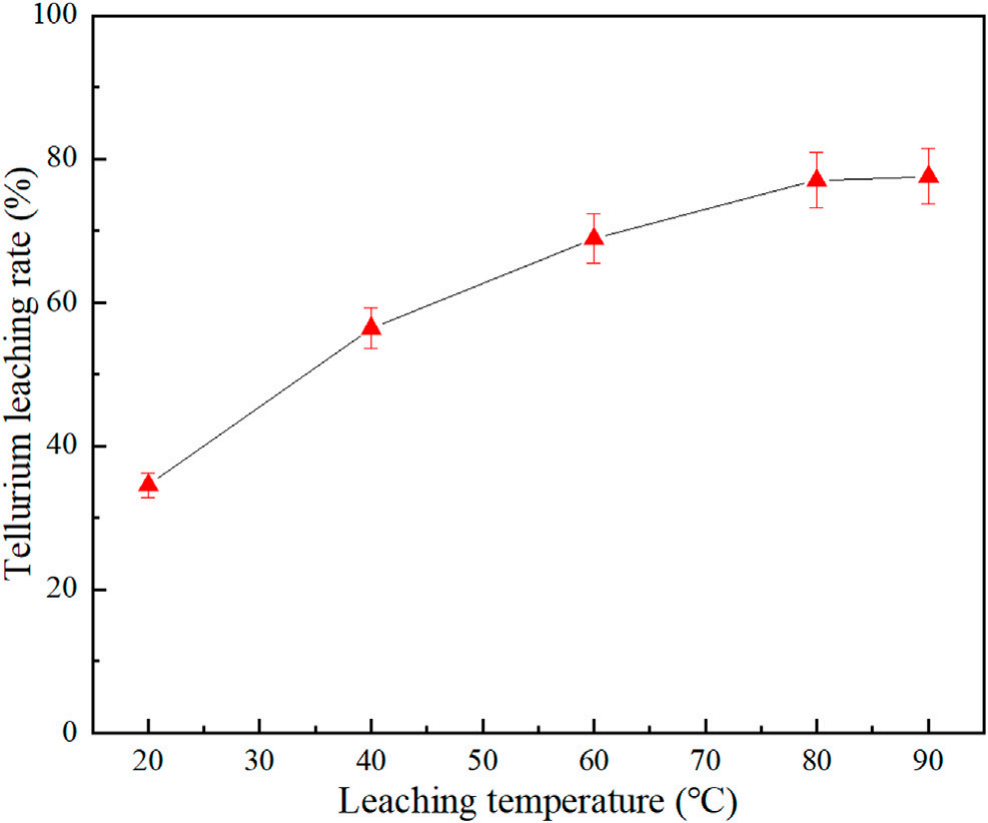
Effect of leaching temperature on tellurium leaching rate.

Figure 6 shows the effect of leaching temperature on the Na_2_S + NaOH cooperative leaching process. The experiment was conducted under the conditions of −0.038 mm (95%) grinding fineness, 80 g/L Na_2_S concentration, 30 g/L NaOH concentration, 4:1 liquid–solid ratio and 4 h′s leaching time.

As shown in Figure 6, the tellurium leaching rate increased significantly with leaching temperature increasing from 20 to 80°C, and the leaching rate of tellurium increased from 34.48 to 77.03%. However, when the temperature continued to increase beyond 80°C, the tellurium leaching rate remained almost unchanged. When the temperature is low, the reaction is controlled by diffusion; when the temperature reaches a set value, the diffusion rate is faster, and the leaching process is converted to chemical control. At this time, the increase in temperature has little effect on the leaching rate, which is consistent with the experiment results. So the reaction temperature was determined at 80°C.

### Effect of Leaching Time

**FIGURE 7:**
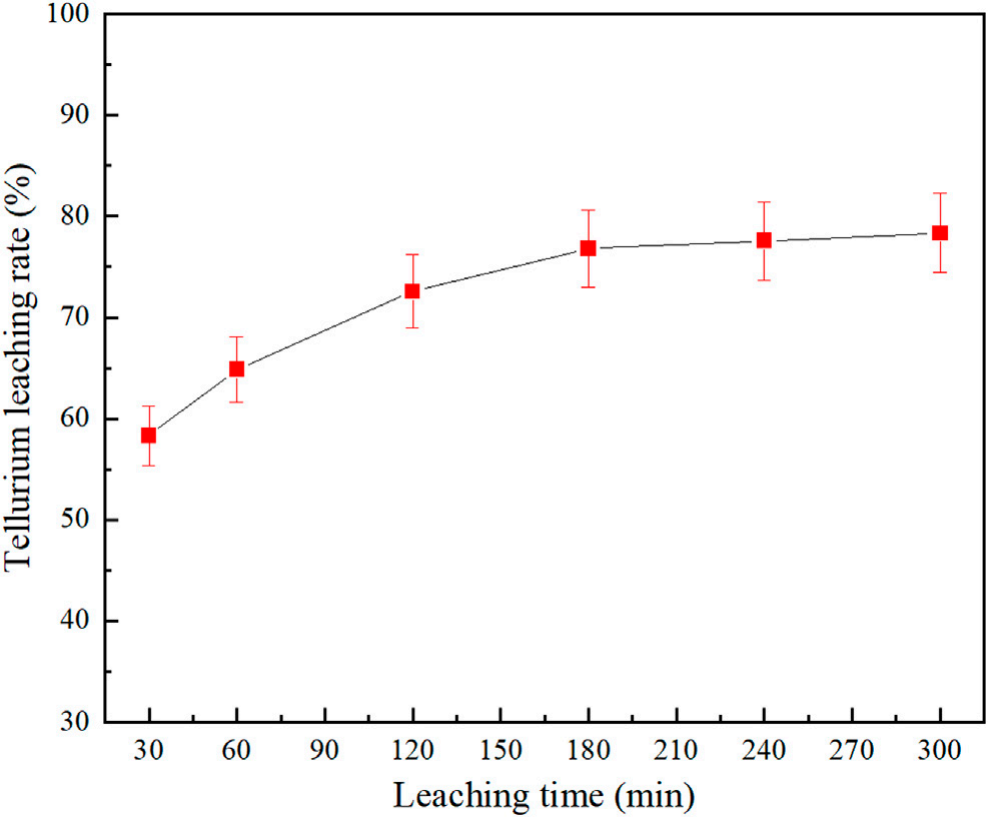
Effect of leaching time on tellurium leaching rate.

Figure 7 shows the effect of leaching time on the Na_2_S + NaOH cooperative leaching process. The experiment was carried out as the grinding fineness was set at −0.038 mm (95%), Na_2_S concentration at 80 g/L, NaOH concentration at 30 g/L, leaching temperature at 80°C and liquid-solid ratio at 4:1.

From Figure 7, it can be seen that the tellurium leaching rate increases with increasing the reaction time and it would come to a stable state. The leaching rate could reach 58.31% at 0.5 h; but when the time was lengthened from 2 to 3 h, the tellurium leaching rate would only increase by 4.26%; then, when the leaching time was beyond 3 h, the tellurium leaching rate would remain almost unchanged. A longer leaching time would facilitate a more thorough leaching process, but it would increase the cost and be detrimental to production, so it was determined that the leaching time was 3 h, at which the tellurium leaching rate was 76.83%.

### Reaction Mechanism Analysis

**FIGURE 8:**
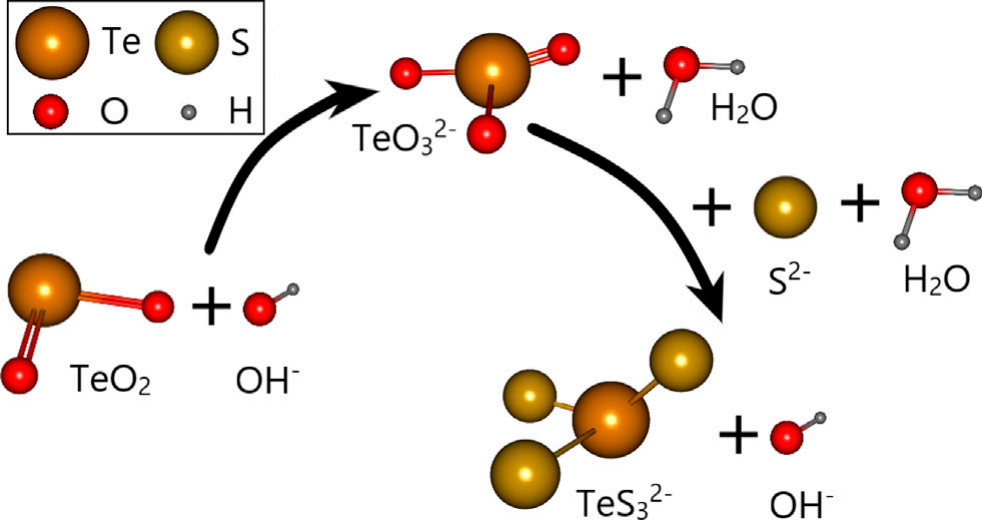
Na_2_S + NaOH cooperative leaching mechanism.

Tellurium is converted to soluble thio-tellurite ($\mathrm{Te}S_{3}^{2-},\mathrm{Te}S_{4}^{2-}$) when tellurium-bearing minerals are dissolved in Na_2_S + NaOH solution (Zhou and Chen, 2008; Liu et al., 2020; Guo et al., 2017). The reaction is shown in Eqs 4–6 and the mechanism diagram is shown in Figure 8. In addition to adjusting the pH value, the addition of NaOH can also effectively prevent the hydrolysis of Na_2_S (Eqs 10,11).

$$Na_{2}S+H_{2}O=\mathrm{NaHS}+\mathrm{NaOH}$$ (10)

$$\mathrm{NaHS}+H_{2}O=H_{2}S+\mathrm{NaOH}$$ (11)

Pb in the telluride-type gold concentrate mainly exists in the form of galena (PbS), and PbS can be leached by NaOH to form soluble lead salt (Eq. 12). (Huang, 2012) The reaction (Eq. 12) caused the consumption of NaOH, thus affecting the leaching of tellurium. The addition of Na_2_S inhibited the reaction (Guo, et al., 2016; Lewis, 2010), and then achieved the selective leaching of tellurium by Na_2_S + NaOH cooperative leaching process. The above mechanism may be further verified through optimum and verification experiments.

$$Na_{2}\mathrm{Pb}O_{2}+Na_{2}S+2H_{2}O=\mathrm{PbS}+4\mathrm{NaOH}$$ (12)

### Optimum Experiment

**FIGURE 9:**
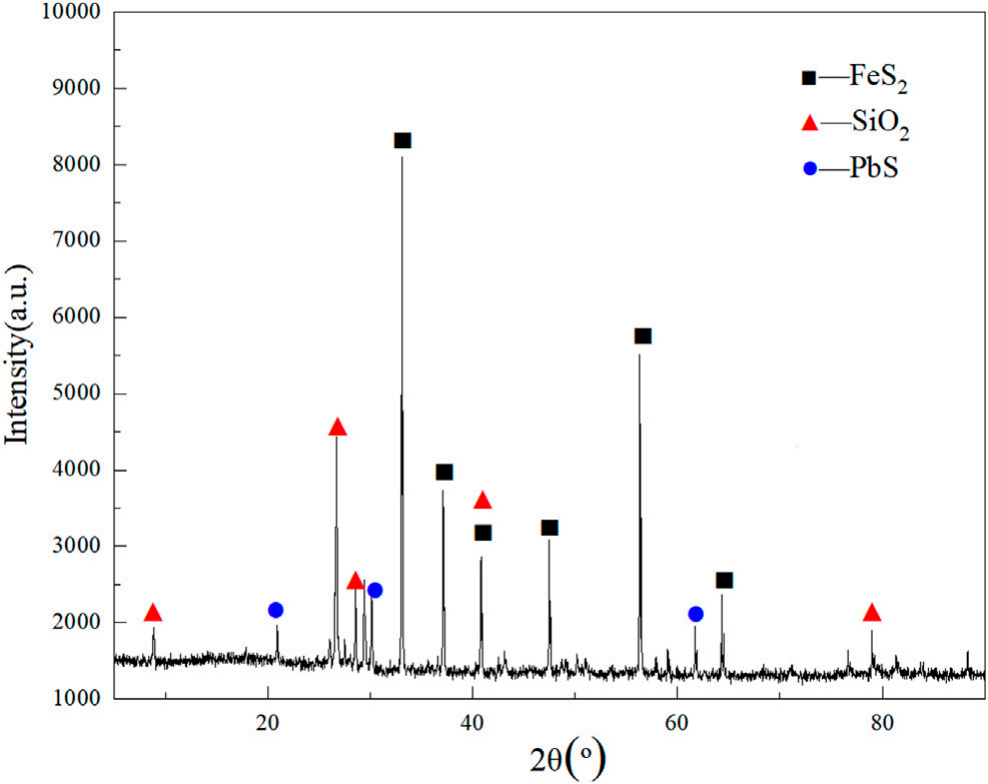
XRD diffractograms of optimum experiment leaching residue.

Through the above experiments, optimum conditions could be obtained for the Na_2_S + NaOH cooperative leaching of tellurium from telluride-type gold concentrate: −0.038 mm (95%) grinding fineness, 80 g/L Na_2_S concentration, 30 g/L NaOH concentration, 80°C leaching temperature, 4:1 liquid-solid ratio, 3 h′s leaching time. The leaching rate of tellurium under these conditions was 78.14% as listed in Table 2. XRD analysis results of the leached slag were performed as shown in Figure 9.

**TABLE 2:** Results of an optimum experiment.

Elemental	Te	Au	Ag	Pb
Leaching residue grade/(g/t)	53.27	86.75	90.14	6.21^a^
Leaching rate/%	78.14	2.86	3.24	8.68

^a^ Means that the unit is %.

From Figure 9, it can be seen that the main phase of the leach residue is still pyrite and that tellurium is not shown in the XRD diffractograms due to its low content. As shown in Table 2, the Pb grade decreased from 6.80 to 6.21%, showing that it was almost unchanged in selective pre-leaching of tellurium from telluride-type gold concentrate. It can be concluded that the leaching of Pb is effectively inhibited by Na_2_S in parallel with tellurium leaching. At the same time, the gold and silver grades are also basically unchanged. It is concluded that the Na_2_S + NaOH cooperative leaching process can selectively leach tellurium from telluride-type gold concentrate, and the possibility of recovering tellurium, gold and silver from telluride-type gold concentrate will be investigated in future experiments.

### Validation Experiment

**FIGURE 10:**
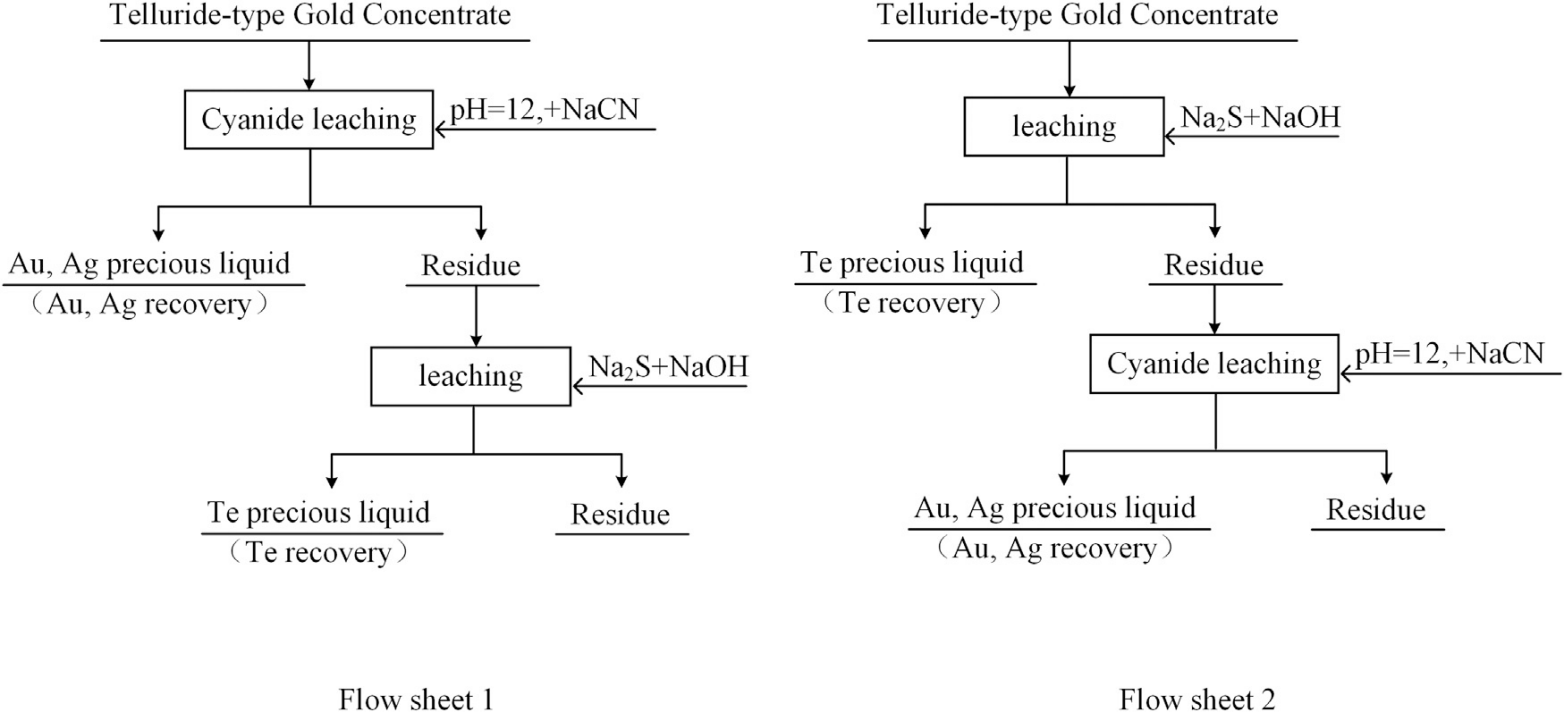
Telluride-type gold concentrate integrated recovery of tellurium, gold, and silver experiment flow chart.

Ores with conventional cyanide leaching gold leach rates of less than 80% are referred to as refractory gold ores (Fraser et al., 1991; Yannopoulos and Springerlink, 1991). The direct leaching gold leaching rate of telluride-type gold concentrate in this experiment was only 32.00%, which is refractory gold ore. To determine the effect of Na_2_S + NaOH cooperative leaching on the recovery of precious metals from telluride-type gold concentrate, a validation experiment was conducted with reference to the flowchart shown in Figure 10, and the experiment results are listed in Table 3.

**TABLE 3:** Results of Validation experiment.

Experimental procedure	Leaching rate/%	
	Au	Ag
1	32.00	50.30
2	75.39	58.83

The pre-leaching experiment of tellurium was carried out as the grinding fineness was set at −0.038 mm (95%), Na_2_S concentration at 80 g/L, NaOH concentration at 30 g/L, leaching temperature at 80°C, liquid-solid ratio at 4:1, and leaching time at 3 h. The leaching of gold and silver was carried out under the following conditions: the grinding fineness was set at −0.038 mm (95%), pH = 12, NaCN dosage at 0.80%, liquid-solid ratio at 2:1, and leaching time at 48 h.

It can be seen from Table 3 that the experiment results of procedure 2 are significantly better than that of procedure 1. When the telluride-type gold concentrate is directly cyanide leached to leaching gold and silver, the gold leaching rate is only 32.00%. After the telluride-type gold concentrate is selectively pre-leached with tellurium, the leaching rate of gold is significantly increased to 75.39%, and the leaching rate of silver also rises to 58.83%, indicating that the hydration film formed by tellurium can interfere with gold and silver leaching, especially gold leaching during the cyanidation process of telluride-type gold mines (Jayasekera et al., 1996; Henley et al., 2001; Dyer et al., 2017). The selective pre-leaching of tellurium before the cyanide leaching of telluride-type gold concentrate can separate and enrich tellurium and effectively improve the leaching rate of conventional cyanidation of precious metals of this type of ore. However, the leaching rates of tellurium, gold and silver are still low. In future research, it hopes to obtain better indicators by optimizing conditions and strengthening leaching, so as to realize the comprehensive recovery of tellurium, gold, and silver in telluride-type gold concentrate.

### Leaching Kinetic Analysis

According to the experimental data at different temperatures, the applicability of diffusion control, interfacial chemical reaction control and mixing control are discussed based on the kinetic equations of equations (Eqs 13, 14). There are many reaction models used in leaching kinetics, including the diffusion-controlled model (Eq. 13) and the chemical reaction-controlled model (Eq. 14) (Demirkıran and Künkül, 2007):

$$-\mathrm{In}\left(1-\varepsilon \right)=\;K_{1}t$$ (13)

$$\frac{1}{1-\varepsilon }-1=K_{2}t$$ (14)

**FIGURE 11:**
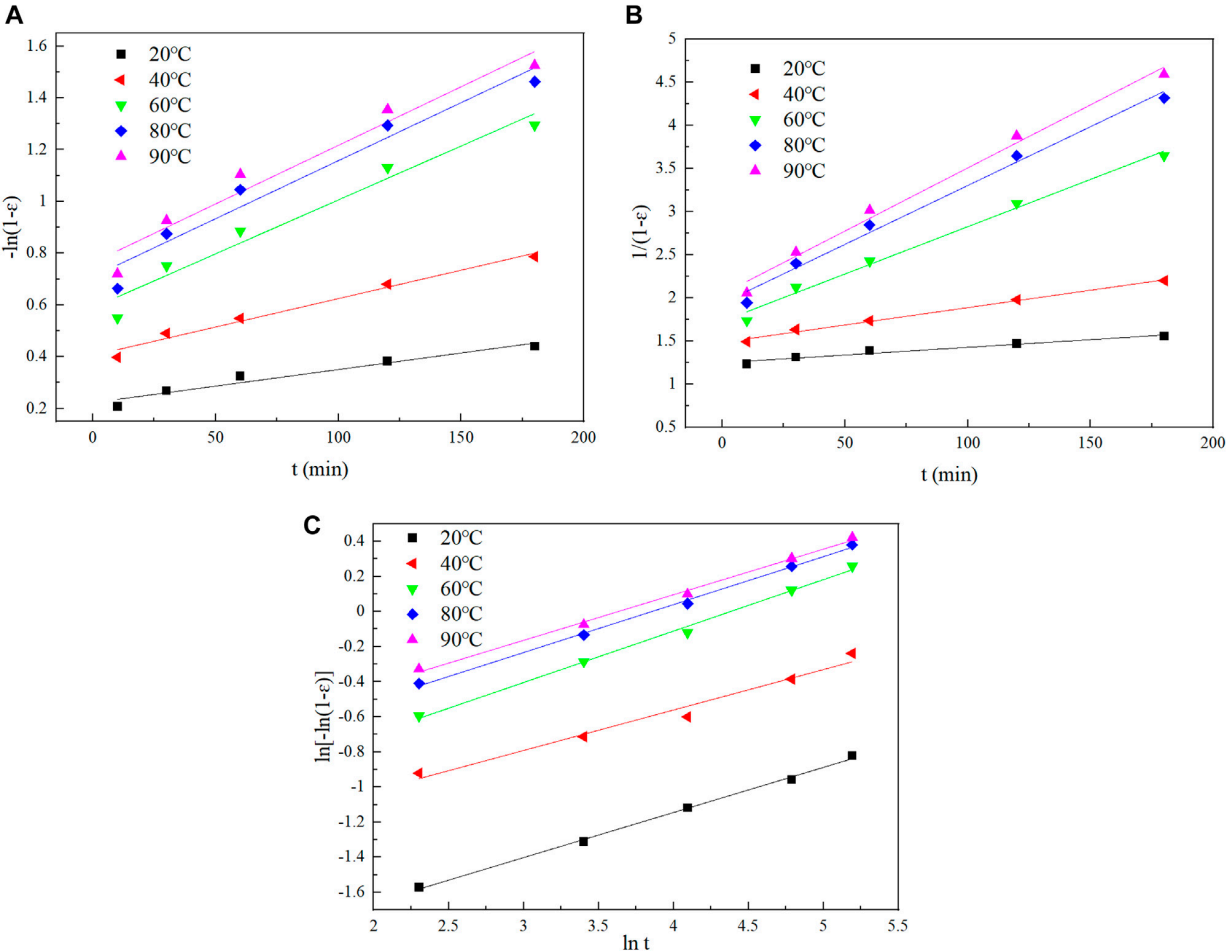
The fitting curves using diﬀerent models: **(A)** the plot of [−ln (1−*ε*)] vs. t, **(B)** the plot of 1/(1−*ε*) vs. t, **(C)** the plot of ln [−ln (1−*ε*)] vs. ln*t*.

In the equations, *ε* is leaching rate, *t* leaching time, and $K_{1},K_{2}$ apparent reaction constants. The plots of linear fitting are shown in Figure 11 and the parameters are listed in Table 4.

**TABLE 4:** Fitting results of the two models.

T/°C	Kinetic expression	Regression equation	Correlation coefficient *R* ^2^
20	$-In\left(1-\varepsilon \right)=K_{1}t$	−In(1−ε)=0.22323+0.00128t	0.94655
	$\frac{1}{1-\varepsilon }-1=K_{2}t$	$\frac{1}{1-\varepsilon }-1=1.24656+0.00179K_{2}t$	0.96069
	$-\ln\left(1-\varepsilon \right)=Kt^{n}\;$	ln[−ln(1−ε)]=−2.17242+0.25693⁡ln⁡t	0.99777
40	$-In\left(1-\varepsilon \right)=K_{1}t$	−In(1−ε)=0.40573+0.00219t	0.98249
	$\frac{1}{1-\varepsilon }-1=K_{2}t$	$\frac{1}{1-\varepsilon }-1=1.48301+0.00403t$	0.99299
	$-\ln\left(1-\varepsilon \right)=Kt^{n}\;$	ln[−ln(1−ε)]=−1.48257+0.23039⁡ln⁡t	0.97555
60	$-In\left(1-\varepsilon \right)=K_{1}t$	−In(1−ε)=0.58977+0.00416t	0.96086
	$\frac{1}{1-\varepsilon }-1=K_{2}t$	$\frac{1}{1-\varepsilon }-1=1.72839+0.01096t$	0.99075
	$-\ln\left(1-\varepsilon \right)=Kt^{n}\;$	ln[−ln(1−ε)]=−1.28314+0.29295⁡ln⁡t	0.99578
80	$-In\left(1-\varepsilon \right)=K_{1}t$	−In(1−ε)=0.70968+0.00448t	0.95396
	$\frac{1}{1-\varepsilon }-1=K_{2}t$	$\frac{1}{1-\varepsilon }-1=1.9378+0.01364t$	0.98927
	$-\ln\left(1-\varepsilon \right)=Kt^{n}\;$	ln[−ln(1−ε)]=−1.05302+0.27317⁡ln⁡t	0.99764
90	$-In\left(1-\varepsilon \right)=K_{1}t$	−In(1−ε)=0.76434+0.00452t	0.95537
	$\frac{1}{1-\varepsilon }-1=K_{2}t$	$\frac{1}{1-\varepsilon }-1=2.04364+0.01462t$	0.98998
	$-\ln\left(1-\varepsilon \right)=Kt^{n}\;$	ln[−ln(1−ε)]=−0.94319+0.25972⁡ln⁡t	0.99651

From the fitting curve and related parameters, the diffusion-controlled model could not describe the leaching process of Te at different temperatures. On the other hand, the chemical reaction-controlled model fitted the tellurium leaching process well. In order to obtain a better fitting effect, the Avrami model was applied (Eq. 15). The results of linear fitting are shown in Figure 11 and Table 4.

$$-\ln\left(1-\varepsilon \right)=Kt^{n}$$ (15)

When 0.5 ≤ *n* < 1, the leaching process of Te is a mixed type of chemical reaction control and diﬀusion control (Gu et al., 2019). In Table 4, the average of *n* values is 0.26263, and it shows the reaction rate is controlled by diffusion at the beginning of the experiment, which is consistent with the experimental data.

The leaching rate can be calculated by the Arrhenius formula:

$$K=Ae^{-\frac{E}{\mathrm{RT}}}$$ (16)

$$\ln k=–\frac{E}{RT}+\ln A$$ (17)

**FIGURE 12:**
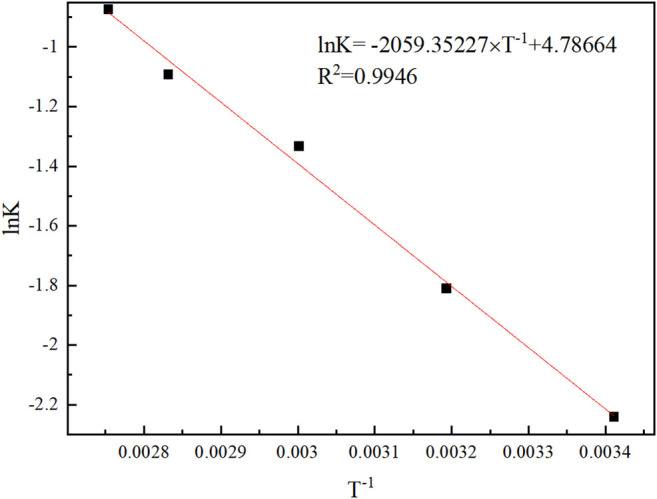
The relation between lnK and 1/T.

*K* is the reaction rate constant, *E* (kJ/mol) the reaction activation energy, *R* (J/mol·k) the ideal gas constant, *T* (Kelvins) the absolute temperature, and *A* the frequency factor. lnA is a constant. Figure 12 is drawn based on the above results. Therefore, the values of E and A are calculated to be 17.12 kJ/mol and A = 119.90.

## Conclusion

The optimum process conditions for the tellurium leaching from Na_2_S + NaOH were determined by investigating the effect of various factors on the tellurium leaching rate in the process of Na_2_S + NaOH cooperative leaching of tellurium from tellurium telluride-type gold concentrate: −0.038 mm (95%) grinding fineness, 80 g/L Na_2_S concentration, 30 g/L NaOH concentration, 80°C leaching temperature, 4:1 liquid-solid ratio. Under these conditions, the leaching rate of tellurium is 78.14%; the leaching rates of gold and silver are both less than 3.5%; the lead content before and after leaching is only reduced by 0.59%. The dates have shown the realization of the selective leaching of tellurium.

The effect of selective pre-leaching of tellurium by Na_2_S + NaOH on the recovery of precious metals from telluride-type gold concentrate was determined by validation experiments. The tellurium leaching conditions remained unchanged, while the gold and silver leaching conditions were as follows: −0.038 mm (95%) grinding fineness, pH = 12, NaCN dosage 0.80%, liquid-solid ratio 2:1, leaching time 48 h. Under these conditions, the leaching rates of Au and Ag in the experiment results of procedure 2 (pre-leaching tellurium) are 75.39 and 58.83%, respectively, which are 43.38 and 8.53% higher than those of procedure 1 (direct cyanide leaching of gold and silver). It effectively eliminates the adverse effects of tellurium on the recovery of precious metals in telluride-type gold concentrate.

The selective leaching of tellurium from telluride-type gold concentrate by the Na_2_S + NaOH cooperative leaching process has provided new ideas for the separation and extraction of tellurium, a rare element, from telluride-type gold concentrate. And pre-leaching tellurium before cyanide leaching can improve the leaching rates of gold and silver, which provides a theoretical and technological basis for the comprehensive recovery of tellurium, gold, and silver from telluride-type gold concentrate.

The kinetic analysis showed that the Na_2_S + NaOH leaching process was in accordance with the diﬀusion-controlled type in the Avrami model, and the leaching of tellurium was clarified to mixed control type. The grain parameter in the leaching process was 0.26263 and the apparent activation energy E = 17.12 kJ/mol.

## Data Availability

The raw data supporting the conclusion of this article will be made available by the authors, without undue reservation.
